# Constructing a question bank based on script concordance approach as a novel assessment methodology in surgical education

**DOI:** 10.1186/1472-6920-12-100

**Published:** 2012-10-24

**Authors:** Salah A Aldekhayel, Nahar A ALselaim, Mohi Eldin Magzoub, Mohammad M AL-Qattan, Abdullah M Al-Namlah, Hani Tamim, Abdullah Al-Khayal, Sultan I Al-Habdan, Mohammed F Zamakhshary

**Affiliations:** 1 Plastic Surgery Division, King Saud bin Abdulaziz University for Health Sciences (KSAU-HS), Riyadh, Saudi Arabia; 2 Plastic Surgery Division, King Saud University, Riyadh, Saudi Arabia; 3 Department of Medical Education, King Saud bin Abdulaziz University for Health Sciences (KSAU-HS), Riyadh, Saudi Arabia; 4 Pediatric Surgery Division, King Saud bin Abdulaziz University for Health Sciences, Riyadh, Saudi Arabia; 5 King Abdullah International Medical Research Center, King Saud bin Abdulaziz University for Health Sciences, Riyadh, Saudi Arabia; 6 Assistant Deputy, Minister of Health for Planning and Training, Saudi Arabia

**Keywords:** Plastic surgery, Script concordance approach, Question bank, Surgical education

## Abstract

**Background:**

Script Concordance Test (SCT) is a new assessment tool that reliably assesses clinical reasoning skills. Previous descriptions of developing SCT-question banks were merely subjective. This study addresses two gaps in the literature: 1) conducting the first phase of a multistep validation process of SCT in Plastic Surgery, and 2) providing an objective methodology to construct a question bank based on SCT.

**Methods:**

After developing a test blueprint, 52 test items were written. Five validation questions were developed and a validation survey was established online. Seven reviewers were asked to answer this survey. They were recruited from two countries, Saudi Arabia and Canada, to improve the test’s external validity. Their ratings were transformed into percentages. Analysis was performed to compare reviewers’ ratings by looking at correlations, ranges, means, medians, and overall scores.

**Results:**

Scores of reviewers’ ratings were between 76% and 95% (mean 86% ± 5). We found poor correlations between reviewers (Pearson’s: +0.38 to −0.22). Ratings of individual validation questions ranged between 0 and 4 (on a scale 1–5). Means and medians of these ranges were computed for each test item (mean: 0.8 to 2.4; median: 1 to 3). A subset of test items comprising 27 items was generated based on a set of inclusion and exclusion criteria.

**Conclusion:**

This study proposes an objective methodology for validation of SCT-question bank. Analysis of validation survey is done from all angles, i.e., reviewers, validation questions, and test items. Finally, a subset of test items is generated based on a set of criteria.

## Background

Research concerning the assessment of clinical reasoning skills has been extensive in the last few decades [1]. Kreiter et al [2] suggest three potentially measurable aspects related to clinical reasoning: (1) to assess whether important information was collected and retained by the physician; (2) to assess diagnosis and management outcomes resulting from the integration of new clinical information with preexisting knowledge structures; and (3) to assess the development of those preexisting knowledge structures. According to Kreiter [2], the script concordance test (SCT), which was described originally by Charlin and collaborators in 2000 [3], is one method that reliably assesses those aspects of clinical reasoning. It has emerged from two theories of clinical reasoning: hypothetico-deductive and illness script theories [4,5]. The hypothetico-deductive theory implies that when physicians encounter a problem in a real-life setting (a diagnostic, investigative, or therapeutic problem), they generate multiple preliminary hypotheses and then test each one to confirm or eliminate these hypotheses until a final decision is reached [6,7]. The illness script theory provides one way of explaining this concept. It indicates that knowledge is organized in networks and that when a new situation is faced, one would activate prior networks to make sense of this new situation [6,8,9]. Schmidt et al [10] elaborate that these scripts emerge from expertise and hence are refined with experience as each new encounter is compiled into relevant mental networks.

The script concordance test (SCT) was designed to probe whether the organization of knowledge networks allows for competent decision-making processes [3]. It places the examinees in a written and authentic environment that resembles their real-life situations. It is based on the following principles [3,11-14]: (1) tasks should be challenging even for experts but still appropriate for the examinees’ level; (2) items should reflect authentic clinical situations and be presented in a vignette format; (3) each item is composed of a clinical scenario and followed by 3–5 questions related to diagnostic, investigative, or management problems; (4) judgments are measured on a 5-point Likert scale for each question; and (5) test scoring is based on an aggregate scoring method.

Over the last decade, extensive research has been conducted that confirms the validity and reliability of the SCT in various medical disciplines. However, to the best of our knowledge, the validity of the SCT has not yet been examined in plastic surgery, which is known for its controversies and uncertainty; therefore, clinical reasoning is a fundamental cornerstone in the assessment of plastic surgery residents.

Downing [15] discussed five sources of validity evidence based on the Standards for Educational and Psychological Testing [16]: (1) content; (2) response process; (3) internal structure; (4) relationship to other variables; and (5) consequences. The current study aims to assess the content source of validity for two reasons: (i) not all sources of validity evidence are required in all assessments [15]; and (ii) at this phase of question bank construction, we do not have any sources of evidence other than the content validity. Other sources of validity evidence (e.g., internal structure and response process) can be assessed after applying this test to plastic surgery residents in the third phase.

All previous studies [3,12,13,17-20] that examined the validity of the SCT have provided a brief description of question bank construction and a merely subjective method of validating it. Therefore, the present study aims to propose a novel objective methodology for the construction of a question bank in plastic surgery based on the script concordance approach, which will help in standardizing the test writing process of SCT across various disciplines. The construction of the SCT comprises three successive phases: (1) the construction and validation of a question bank; (2) the establishment of a scoring grid; and (3) the application of the test to examinees. This study represents the first phase: question bank construction. Subsequent phases will be conducted in future studies.

## Methods

A validation study was conducted at King Saud bin Abdulaziz University for Health Sciences, Riyadh, between July 2009 and December 2010. The test blueprint (Table 1) was designed to represent the major domains of the plastic surgery residency training program objectives of the Saudi Commission for Health Specialties (SCFHS) and the Royal College of Physicians and Surgeons of Canada (RCPSC).

**Table 1:** Test blueprint

**Topics**	**Number of items**
Pediatric Plastic Surgery	9
Hand Surgery	10
Aesthetic Surgery	7
Breast Surgery	4
Craniofacial Surgery	4
Peripheral Nerves	8
Burn	2
Reconstructive Surgery	8
Total	52

### Item construction

The first step in writing the test items was to invite two academic plastic surgeons at King Saud University and King Saud bin Abdulaziz University for Health Sciences, Riyadh, to develop a pool of real-life clinical scenarios for use in the SCT. They answered the following questions: (i) describe authentic clinical situations that contain an element of uncertainty; (ii) specify for each situation: a) relevant hypotheses, investigation strategies, or management options; b) questions they ask when taking a patient history, signs they look for during the physical examination, and tests that they order to solve the problem; and c) clinical information, whether positive or negative, they would look for in these queries [3]. Multiple drafts were generated and revised until the test writers have reached consensus on the final draft.

**Figure 1:**
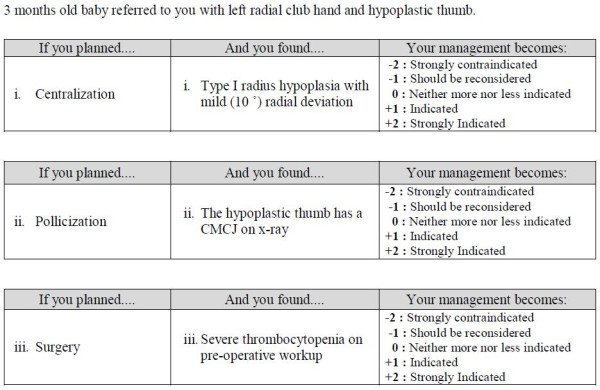
A sample of a script concordance test item composed of a vignette followed by three questions related to management problems.

Next, 52 test items were written by the test writers based on the key features concept [3]. Each item consisted of a vignette followed by two to four questions related to diagnosis, investigation, and/or management, which yielded the first question bank draft with 158 questions. The questions were written in the SCT format in three columns; the first column provides an initial hypothesis, the second column gives a new clinical information (such as a symptom, a sign, a lab result, an imaging result, etc.), and the last column provides a 5-point Likert scale to judge the effect of the new information on the initial hypothesis (Figure 1). The Likert scale ranges between −2 and +2, where −2 or −1 anchors represent a negative effect, +2 or +1 anchors represent a positive effect, and zero anchor represents neither a positive nor a negative effect.

### Item validation

**Figure 2:**
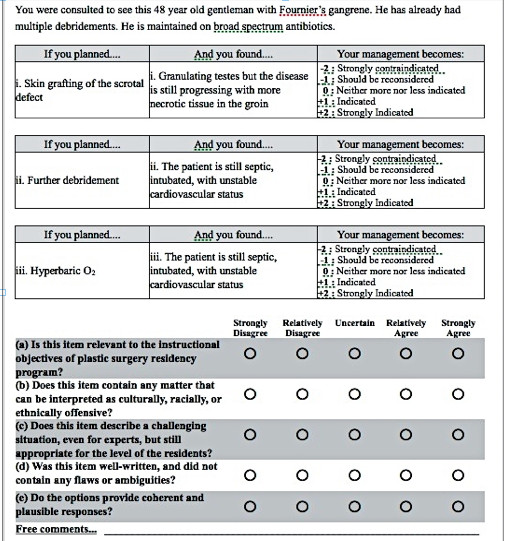
A sample of a SCT item followed by five validation questions that were rated by the reviewers.

Five validation questions (VQ) were developed based on the Standards for Educational and Psychological Testing (prepared by the Joint Committee on Standards for Educational and Psychological Testing of the American Educational Research Association, the American Psychological Association, and the National Council on Measurement in Education) [16]. These questions cover five main areas of content validity (Figure 2): (VQ1) relevance to training program objectives; (VQ2) cultural sensitivity; (VQ3) structural quality of test questions; (VQ4) written clarity of questions; and (VQ5) plausibility of provided options.

A validation survey (Figure 2) was established on the online survey software, SurveyMonkey™, to validate the question bank draft and the test blueprint. The survey was sent to seven academic plastic surgeons in Riyadh, Saudi Arabia and Toronto and Montreal, Canada who met the following inclusion criteria: (1) to be an academic, certified plastic surgeon involved in teaching plastic surgery residents; and (2) have a minimum experience of 10 years in practice. The selection of reviewers was based on a convenient sampling. Ethical approval was obtained from the Institutional Review Board at King Abdullah International Medical Research Center (KAIMRC), Riyadh. All reviewers have agreed to an informed consent before answering the online survey. The survey started by a Likert-type question to rate whether the test blueprint is representative of the educational objectives of Plastic Surgery residency training programs. Then, each test item (clinical scenario followed by 3 to 5 questions) was presented in the survey and followed by the five validation questions described previously (Figure 2).

### The analysis

The analysis of the validation survey was approached from three angles:

(1) Analysis of the reviewers’ ratings:

There were five validation questions for each test item, and each validation question was rated from 1 to 5 (on a 5-point Likert scale from “strongly disagree” to “strongly agree”). For calculation purposes, sum of the ratings of each test item was transformed into a percentage. An overall score represented the average of all reviewers’ scores for each test item. Next, inter-rater reliability was analyzed in terms of correlations between the reviewers’ ratings. Pearson’s coefficients were considered significant at p-value (2-tailed) ≤ 0.05.

(2) Analysis of the validation questions:

Each validation question (VQ) was given a score representing the sum of the ratings by all reviewers on that specific validation question. This score was transformed into a percentage for calculation purposes. Next, ranges of the validation questions were calculated. For any VQ, the maximum possible range was 4, and the minimum was 0 (based on the 5-point Likert scale). The means and medians of these ranges, for each test item, were calculated as well. Differences between the validation questions were considered significant when p-value is ≤ 0.05.

(3) Analysis of the test items:

For ranking purposes, the overall scores of the test items were divided into percentiles: 75^th^, 50^th^, and 25^th^. Then, an item reduction process was carried out to reduce the number of test items from 52 items to a minimum of 20 items. The 20-item SCT was required to achieve a high reliability (Cronbach alpha > 0.75) [12]. This subset of the test items was generated based on a set of inclusion and exclusion criteria which were set arbitrarily and validated with a sensitivity analysis by changing one criterion at a time and looking at the output of these criteria until we reached the optimal end results where the output items have the highest rating. This helps to decrease any margin of error with setting up these criteria arbitrarily. These criteria are:

Inclusion criteria:

□ All items above the 50^th^ percentile (total score ≥ 86%);

□ All items with a mean of the range ≤ 2; and

□All items with a median of the range ≤ 2.

Exclusion criteria:

□ Any item with a range of 4 on any validation question.

These criteria were applied on each domain of the test blueprint separately, as not to disturb the structure of the test. The generated subset of test items will serve as the final draft of the question bank.

Statistical analysis was performed using SPSS version 18 (IBM; Chicago).

## Results

Five out of seven reviewers answered the validation survey completely (response rate 71%): two Saudis and three Canadians. They represented four different academic institutions in Riyadh, Toronto, and Montreal. Regarding the test blueprint (Table 1), three reviewers (60%) were in relative agreement that it was reasonably representative of the major instructional objectives of the plastic surgery residency program, one reviewer (20%) was uncertain, and one (20%) relatively disagreed, suggesting that more burn and reconstruction items must be added. Other comments suggested adding skin pathology as a separate entity in the blueprint, although there were few questions on this subject in the reconstruction domain.

The results of the validation survey are presented under three subheadings: (1) analysis of reviewers’ ratings; (2) analysis of validation questions; and (3) analysis of test items.

(1) Analysis of reviewers’ ratings:

The item scores given by the first reviewer ranged between 40% and 80% (mean 70% ± 10), for the second reviewer between 55% and 100% (mean 96% ± 8.4), for the third reviewer between 50% and 100% (mean 76.8% ± 16.7), for the fourth reviewer between 70% and 100% (mean 94% ± 8), and for the fifth reviewer between 60% and 100% (mean 93% ± 9.5).

Next, we examined the correlations between each reviewer against the average of the remaining reviewers for each validation question and for the overall score (Table 2). Due to the poor overall correlations shown in Table 2, one would assume that potentially one reviewer (or more) is (are) the cause of such poor correlations. Therefore, to confirm or refute this assumption, we repeated the correlations on a pair-by-pair basis, considering one pair of reviewers at a time for every validation question and for the overall score. Pearson’s correlation coefficients fell in the range between +0.38 and −0.22 (p-value > 0.05). Apparently, this process did not reveal any improvement of the correlation.

(2) Analysis of validation questions:

**Table 2:** Pearson correlation coefficients of each reviewer against the average of the remaining reviewers for each validation question (VQ1-VQ5) and for the overall score

	**Reviewer 1**	**Reviewer 2**	**Reviewer 3**	**Reviewer 4**	**Reviewer 5**
**VQ1^**	0.08	CONSTANT	0.07	0.3**	0.21
	P=0.6		P=0.6	P=0.03	P=0.1
**VQ2^**	0.13	0.18	−0.17	CONSTANT	−0.08
	p= 0.4	p= 0.2	p= 0.2		p= 0.6
**VQ3^**	−0.02	0.18	0.01	0.29**	0.05
	p=0.9	p=0.2	p= 0.9	p=0.03	p= 0.8
**VQ4^**	−0.03	−0.01	0.11	0.02	0.24
	p=0.8	p= 0.9	p= 0.4	p=0.9	p= 0.1
**VQ5^**	−0.04	0.07	0	−0.05	0.09
	p= 0.8	p=0.6	p=0.9	p= 0.7	p= 0.5
**Total Score**	−0.03	−0.1	−0.07	0.2	0.15
	p= 0.9	p= 0.5	p= 0.6	p=0.2	p= 0.3

** Correlation is significant at the 0.05 level (2-tailed).

^ VQ denotes “validation question”.

CONSTANT indicates that at least one reviewer has given all test items a constant rating for one of the validation questions.

The scores for the first validation question (VQ1) ranged between 80% and 100% (mean 91% ± 5.6), for VQ2 between 60% and 100% (mean 91% ± 6.7), for VQ3 between 60% and 95% (mean 82% ± 7), for VQ4 between 50% and 95% (mean 81% ± 9.7), and for VQ5 between 60% and 95% (mean 85% ± 6.7).

The ranges of the validation questions are presented in Table 3. The mean of these ranges for the test items fell between 0.8 and 2.4, and the median were between 1 and 3. We observed that 86.5% to 100% of the test items fell within a range of 2 or less for all validation questions except VQ4, for which 71% of the items fell within that range. Therefore, an in-depth analysis was performed specifically for VQ4 to identify the cause of the high variance observed in its range. We hypothesized that a possible underlying cause of such high variance was the different linguistic backgrounds of the reviewers, i.e., Canadians with the English language as their native language, and Saudis with the English language as their second language, keeping in mind that VQ4 asks about the written quality of the test items. Therefore, analysis of VQ4 was repeated after grouping the reviewers into two groups: Saudi and Canadian. The mean of the Saudi scores was 91% ± 13, and the mean of the Canadian scores was 73.5% ± 13.5 (p-value < 0.0001). Furthermore, after recalculation of the ranges of each group individually (Table 4), 92% of the test items in the Saudi group fell within a range ≤ 2; 88.5% of the test items fell within this range in the Canadian group, compared to the initial 71% of test items that fell within the same range before grouping the reviewers (p-value < 0.0001). This means that both groups were homogeneous when considered individually, but when combined they became heterogeneous, which confirmed our hypothesis that the different linguistic backgrounds of the reviewers was the cause of the observed high variance in VQ4.

(3) Analysis of the test items:

**Table 3:** Ranges of validation questions (VQ) ratings

	**Range_VQ1**		**Range_VQ2**		**Range_VQ3**		**Range_VQ4**		**Range_VQ5**	
Min	0		0		0		1		1	
Max	2		4		3		4		4	
Variance	0.36		0.65		0.49		0.75		0.56	
**Range**	**No. of items**	**Cumulative %**	**No. of items**	**Cumulative %**	**No. of items**	**Cumulative %**	**No. of items**	**Cumulative %**	**No. of items**	**Cumulative %**
0	7	13.5	1	1.9	2	3.8	0	0	0	0
1	33	76.5	30	59.6	25	51.9	17	32.7	23	44.2
2	12	100	16	90.4	21	92.3	20	71.2	22	86.5
3	0	100	3	96.2	4	100	13	96.2	6	98.1
4	0	100	2	100	0	100	2	100	1	100

p-value < 0.0001.

**Table 4:** **Ranges of the 4**^**th**^**validation question (written quality of test items) for the Saudi and Canadian groups**

**Range**	**Saudi group**		**Canadian group**	
	**No. of items**	**Cumulative %**	**No. of items**	**Cumulative %**
0	31	59.6	1	1.9
1	14	86.5	30	59.6
2	3	92.3	15	88.5
3	4	100	5	98.1
4	0	100	1	100

p-value < 0.0001.

The overall scores of the test items ranged between 76% and 95% (mean 86% ± 5). These scores were then divided into percentiles: 75^th^ at 90%, 50^th^ at 86%, and 25^th^ at 82%.

The process of subset generation using the inclusion/exclusion criteria yielded 27 eligible items, which are considered to comprise the final draft of the question bank.

## Discussion

The script concordance test was developed in 2000 by Charlin and collaborators [3] who aimed to assess clinical reasoning skills. It places the examinees in a written and authentic environment that resembles their real-life situations. It utilizes an aggregate scoring method that is most suitable for such ambiguous situations [21]. Meterissian [17] indicated that these situations can force a surgeon to deviate from his preoperative plan, and such decisions under pressure could negatively affect patients’ outcomes. Thus, the objective of this study was to address two gaps in the literature: the first goal was to conduct the first phase of a multistep validation study of SCT in the context of plastic surgery, and the second was to provide an objective method to establish and validate a question bank based on the SCT.

The first phase in a multistep validation process constitutes a question bank construction. It comprises four sub-steps: (1) developing a test blueprint; (2) writing test items; (3) validating the question bank draft by external reviewers; and (4) analyzing the validation survey results and generating a subset of the question bank that will be used in the second phase of the SCT validation process, i.e., the establishment of a scoring grid.

Fifty-two test items composed of 158 questions were written, representing the first draft of the question bank. Gagnon et al [22]. found that a 25-item SCT with 3 questions / item achieved the highest reliability (Cronbach’s alpha > 0.80) with the minimum cognitive demand on examinees (test time of one hour) and a minimal workload for the test writers. However, when constructing the question bank, one must keep in mind that a significant number of items will be discarded or rewritten during the question bank reviewing process and score grid establishment. Meterissian [17] suggested an initial 100-question SCT to provide a margin for the item reduction process. Item reduction occurs at two levels: the first is based on reference panel comments [12], and the second occurs following an analysis of reference panel scores, where items with extreme variability should be discarded [23]. The validation survey enabled us to select the best test items, and according to the set criteria, 27 items composed of 83 questions met those criteria. Moreover, a good margin was obtained for further reduction of the number of items in the second phase (establishing the score grid) while maintaining high reliability (Cronbach’s alpha > 0.75).

The question bank validation process is a crucial step in constructing the SCT. It assures the face validity (whether the questions test clinical reasoning skills) and content validity (whether the questions are relevant and representative of the training program objectives) [20]. For the content validation purposes, we developed five validation questions (Figure 2) examining five different domains: (i) relevance to the training program objectives; (ii) cultural sensitivity; (iii) structural quality of test questions; (iv) written quality of questions; and (v) plausibility of provided options.

The analysis of the validation survey was approached from three angles: reviewers, validation questions, and test items. This was performed to determine whether all elements of the validation process had been examined because any element could be a threat to this process. For instance, one might consider that a reviewer who persistently under- or over-rates test items, or even a poorly written validation question, could affect the validation process if that situation is not taken into consideration and controlled.

The analysis of reviewers’ ratings aimed to identify an agreement between reviewers. Correlations between each reviewer against the pool of the remaining reviewers were poor. Surprisingly, even correlations between paired reviewers were poor. One would assume that such poor correlations could be attributed to any of the following assumptions: (a) small sample size (5 reviewers); (b) poorly written validation questions; (c) heterogeneity of the reviewers, i.e., different cultural and subspecialty backgrounds. However, the validation question analysis did not show a consistently poor VQ; although VQ4 demonstrated a high variability, further in-depth analysis (Table 4) provided an explanation for this finding. It is important to note that the sample size could have had a negative effect; however, we cannot ignore the possibility that the heterogeneity of the reviewers might have been the cause of such poor correlations. We decided to give an equal weight to all reviewers’ ratings, and we generated a subset of test items based on them. One strategy to address such poor correlation is the development of inclusion/exclusion criteria that aim to select the best rated items to be included in the second phase of the validation process (establishing the scoring grid).

This study has few limitations. In addition to the previous discussion concerning the poor correlations between reviewers, certain test items exhibited a high level of disagreement for certain validation questions. For instance, one test item provided one “strongly disagree” rating and four “strongly agree” ratings! These items were eventually excluded from the final draft of the question bank because they did not meet the inclusion criteria, but such an unexplainable disagreement between the reviewers is striking. Another limitation of the study is the lack of accessibility to the reviewers because they are from different institutions and countries. Ideally, a poorly rated test item can be rewritten and resubmitted to the reviewers for revalidation. Other sources of evidence for the construct and criterion validity and reliability will be collected in the future studies as the question bank undergoes the application phase. Finally, although the overall validation process could seem complicated, it enriches the test writers with a validated and objectified methodology to construct SCT-based question banks.

## Conclusions

This study represents the first phase of SCT validation in the context of plastic surgery: the construction of a question bank. It proposes an objective methodology for validation of the question bank. Basically, after experts develop the test blueprint and write test items, a validation survey should be established and then sent to external reviewers. Analysis of the validation survey should be conducted from all possible angles, e.g., reviewers’ correlations, validation questions, and test items. Finally, a subset of test items should be generated based on a set of inclusion and exclusion criteria. Further studies will be conducted to complete the remaining phases of the SCT validation (establishing a score grid and application to plastic surgery residents).

## Competing interest

The authors declare that they have no competing interests.

## Authors’ contributions

SA designed the study; participated in data analysis and manuscript drafting. NA participated in the study design, data analysis and manuscript drafting. AA participated in data analysis, interpretation and study co-ordination. HT participated in data analysis and interpretation. AA helped in study design and reviewed the manuscript. SA participated in the study design and manuscript drafting. MM participated in the study design and manuscript drafting. MZ coordinated the research and made a critical review of the manuscript. All authors read and approved the final manuscript.

## Disclosure

The study has not received financial grants from any institution.

The study is a Master’s thesis done in partial fulfillment of the Masters of Medical Education program at College of Medicine, King Saud bin Abdulaziz University for Health Sciences, Riyadh, Saudi Arabia.

## Pre-publication history

The pre-publication history for this paper can be accessed here:

http://www.biomedcentral.com/1472-6920/12/100/prepub
